# Supplementary material for: Metabolite Profiling of Manilkara zapota L. Leaves by High-Resolution Mass Spectrometry Coupled with ESI and APCI and In Vitro Antioxidant Activity, α-Glucosidase, and Elastase Inhibition Assays
Source: Int J Mol Sci. 2020 Dec 24;22(1):132. doi: 10.3390/ijms22010132 (PMC7795549; doi:10.3390/ijms22010132)
Supplement: Supplementary file 1 [file ijms-22-00132-s001.zip › Supplementary-materials_ZLE_Final.pdf]

Metabolite Profiling of *Manilkara zapota* L. Leaves by High-Resolution Mass Spectrometry Coupled with ESI and APCI and in Vitro Antioxidant Activity, α-Glucosidase, and Elastase Inhibition Assays

Syful Islam [1,2,†], Md Badrul Alam [3,4,†], Hyeon-Jin Ann [3], Ji-Hyun Park [3], Sang-Han Lee [3,4,5,*] and Sunghwan Kim [1,6,*]

[1] Department of Chemistry, Kyungpook National University, Daegu 41566, Korea; msi412@yahoo.com
[2] Department of Environment, Munshiganj District Office, Munshiganj-1500, Bangladesh
[3] Department of Food Science and Biotechnology, Kyungpook National University, Daegu 41566, Korea; mbalam@knu.ac.kr (M.B.A.); jiny345@knu.ac.kr (H.-J.A.); wlgus6744@knu.ac.kr (J.-H.P.)
[4] Food and Bio-Industry Research Institute, Inner Beauty/Antiaging Center, Kyungpook National University, Daegu 41566, Korea
[5] knu BnC, Daegu 41566, Korea
[6] Mass Spectrometry Converging Research Center and Green-Nano Materials Research Center, Kyungpook National University, Daegu 41566, Korea
* Correspondence: sang@knu.ac.kr (S.-H.L.); sunghwank@knu.ac.kr (S.K.)
† These authors have equal contribution

Supplementary materials:

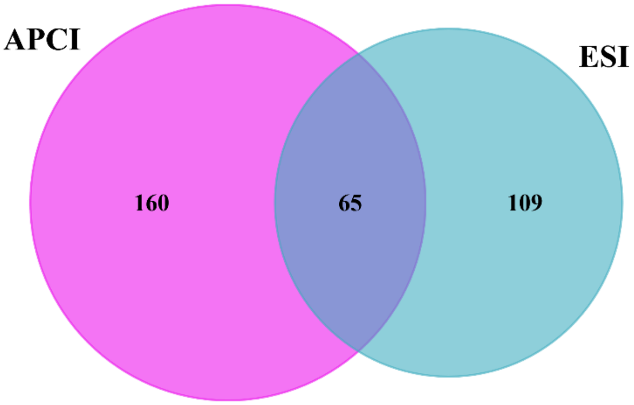

Figure S1: Venn diagram showing the overlapping peaks detected in (−) mode electrospray ionization (ESI) and atmospheric pressure chemical ionization (APCI) mass spectrometry (MS).

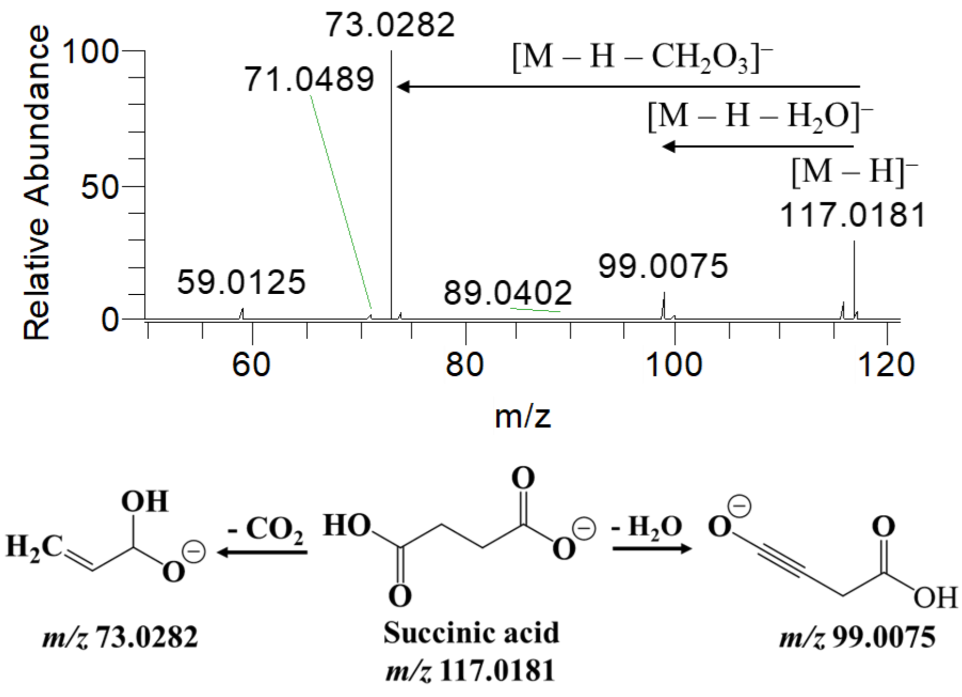

Figure S2: (–) mode electrospray ionization tandem mass spectrometry (ESI MS/MS) fragmentation pattern of succinic acid.

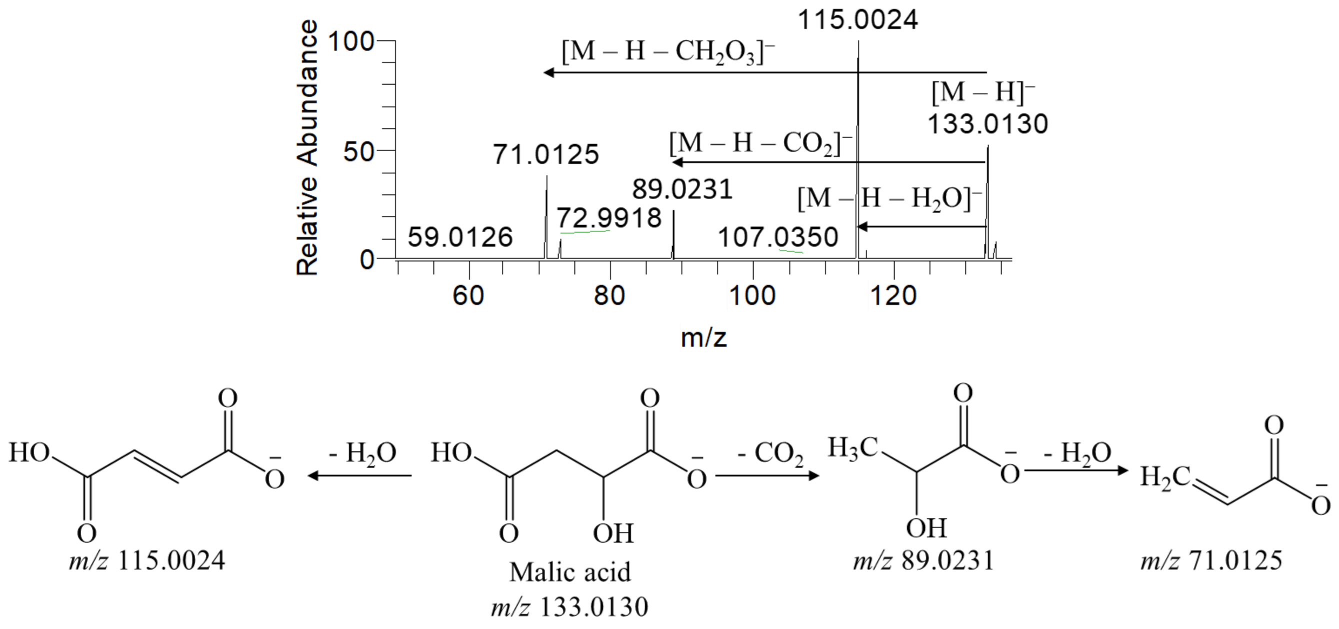

Figure S3: (–) mode electrospray ionization tandem mass spectrometry (ESI MS/MS) fragmentation pattern of malic acid.

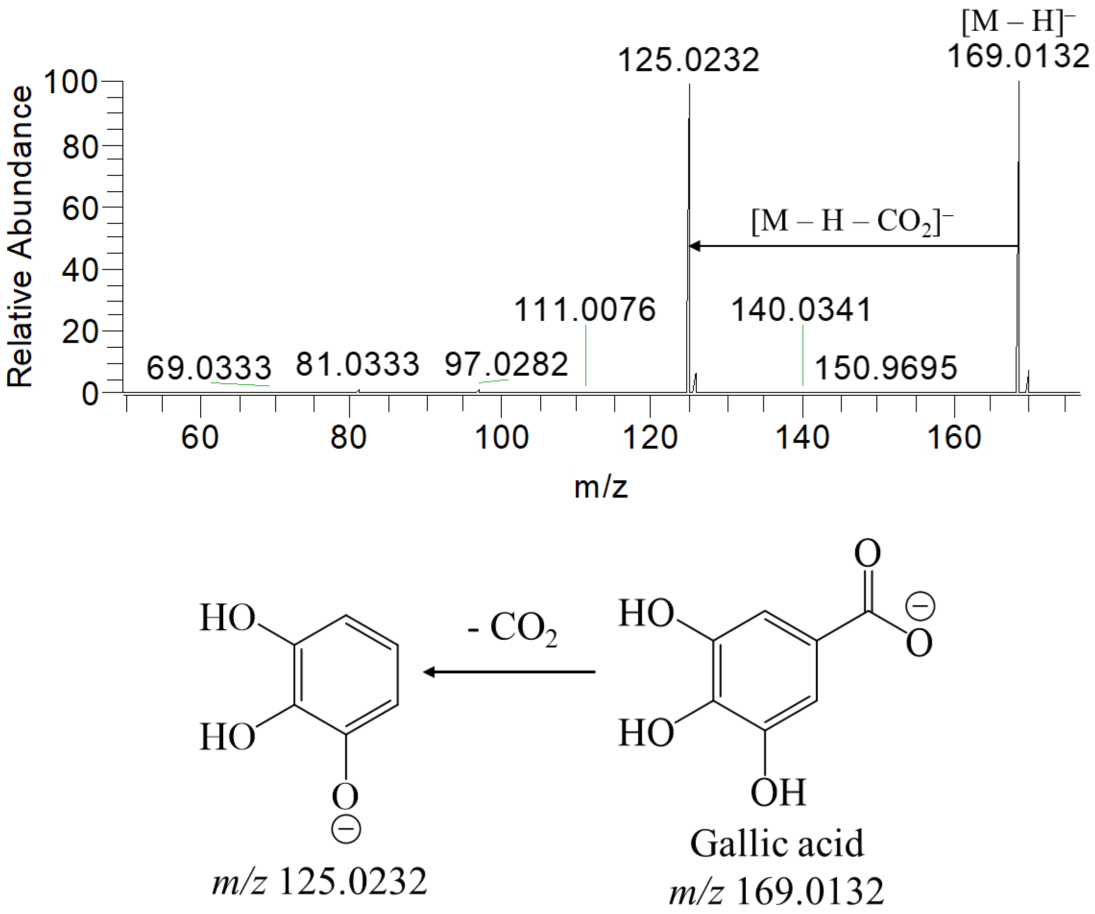

Figure S4: (–) mode electrospray ionization tandem mass spectrometry (ESI MS/MS) fragmentation pattern of gallic acid.

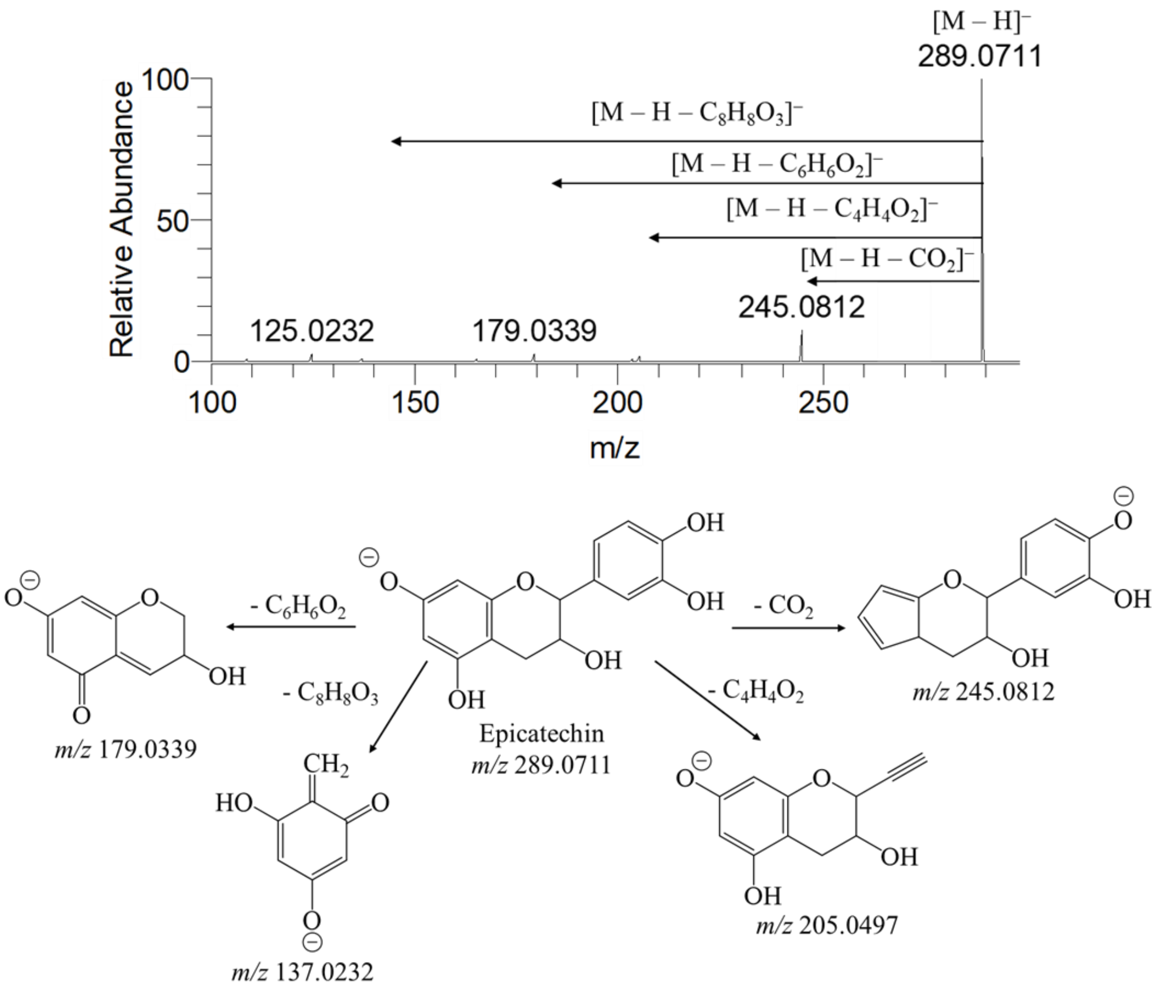

Figure S5: (–) mode electrospray ionization tandem mass spectrometry (ESI MS/MS) fragmentations of epicatechin.

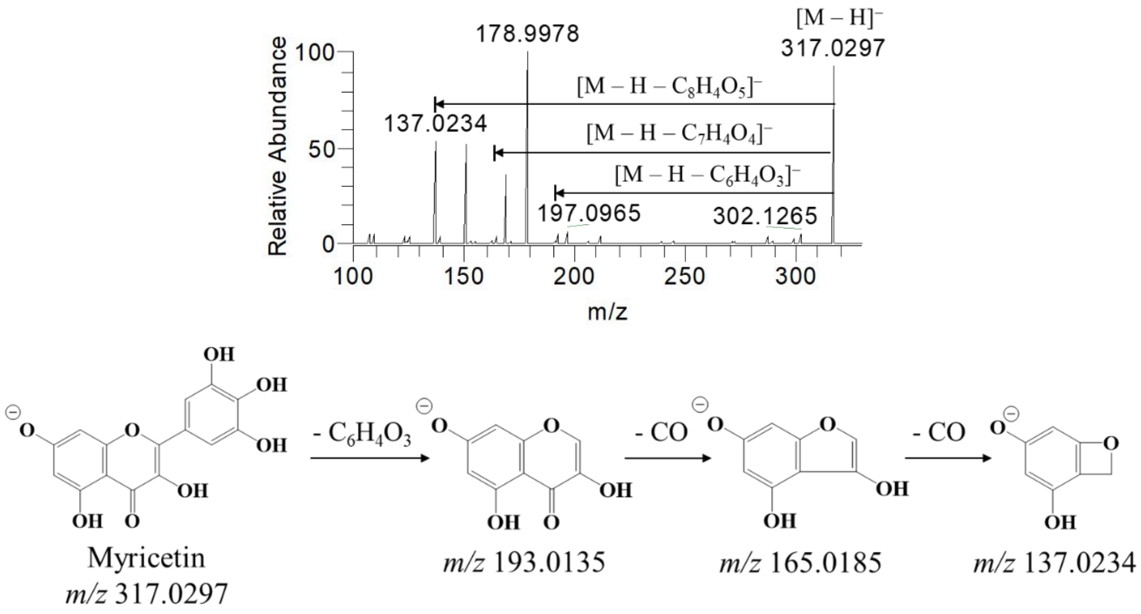

Figure S6: (–) mode atmospheric pressure chemical ionization tandem mass spectrometry (APCI MS/MS) fragments of myricetin.

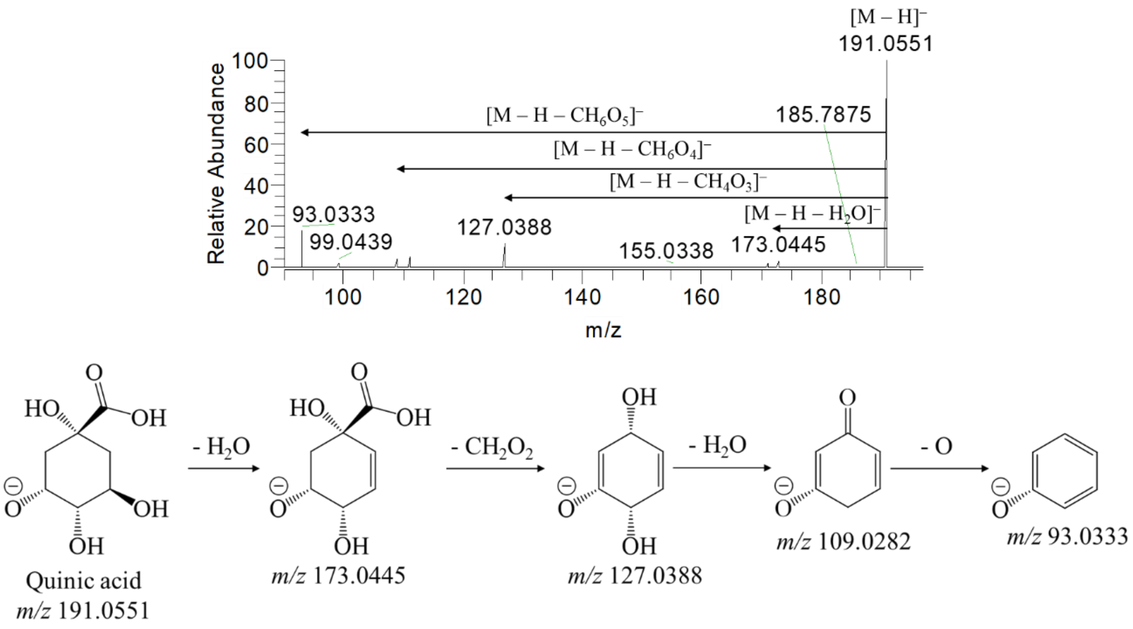

Figure S7: (–) mode electrospray ionization tandem mass spectrometry (ESI MS/MS) fragments of quinic acid.

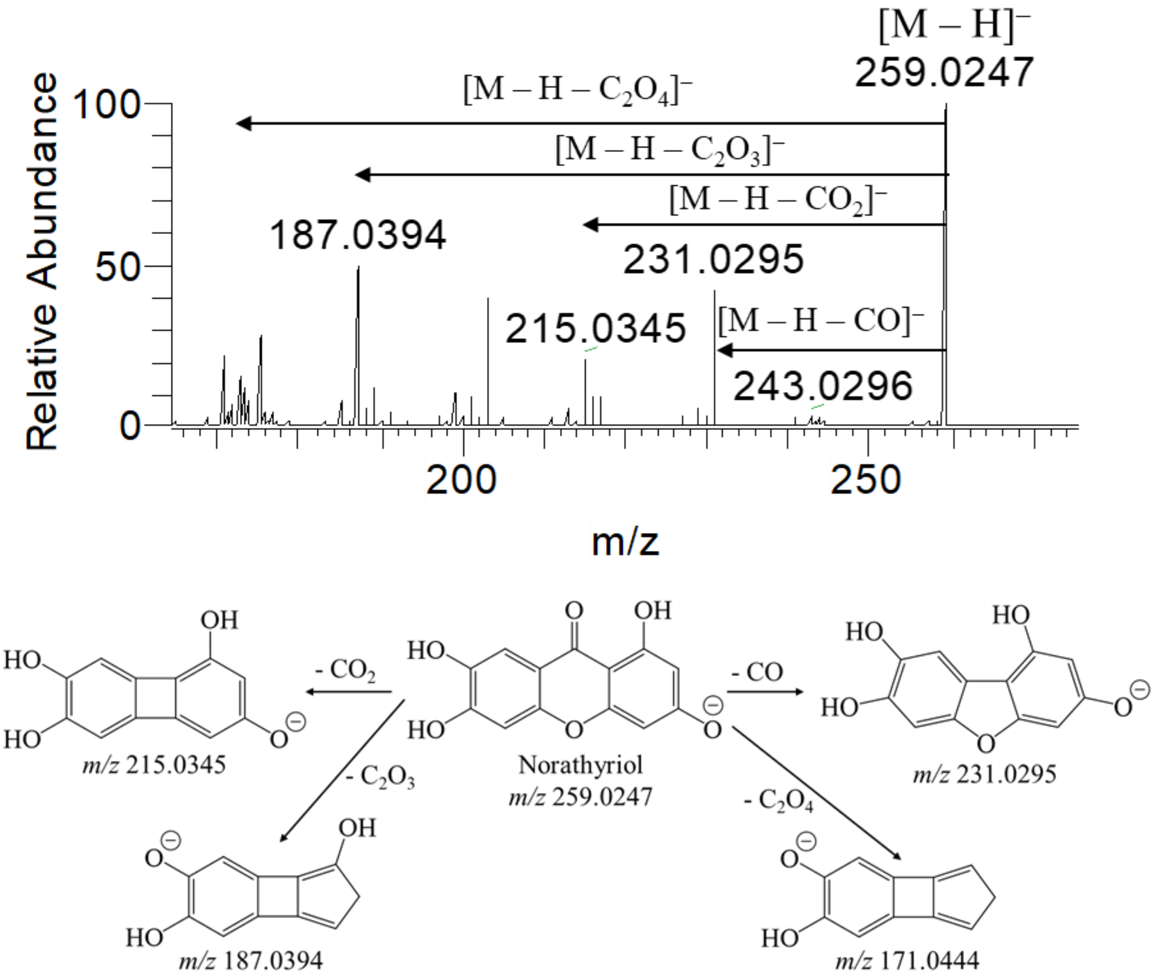

Figure S8: (–) mode atmospheric pressure chemical ionization tandem mass spectrometry (APCI MS/MS) fragments of norathyriol.

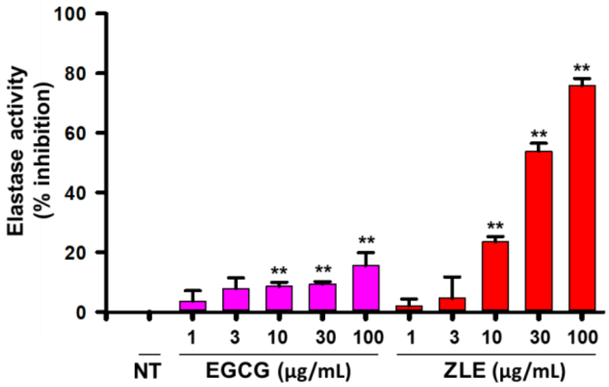

Figure S9: Elastase-inhibition activities of *Manilkara zapota* leaves ethanol extracts (ZLE).

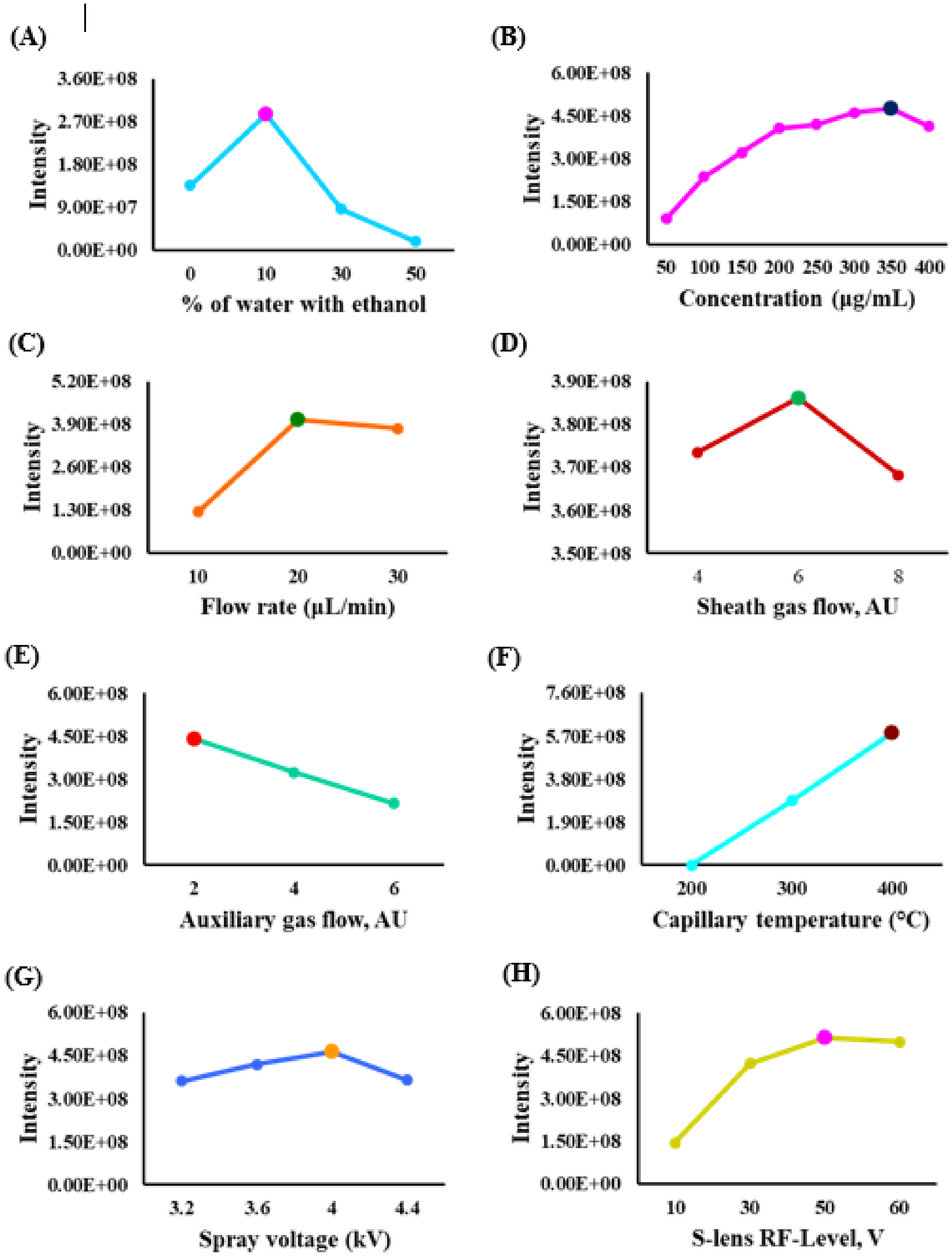

Figure S10: Optimization of negative-mode electrospray ionization mass spectrometry (ESI–MS) operating parameters.

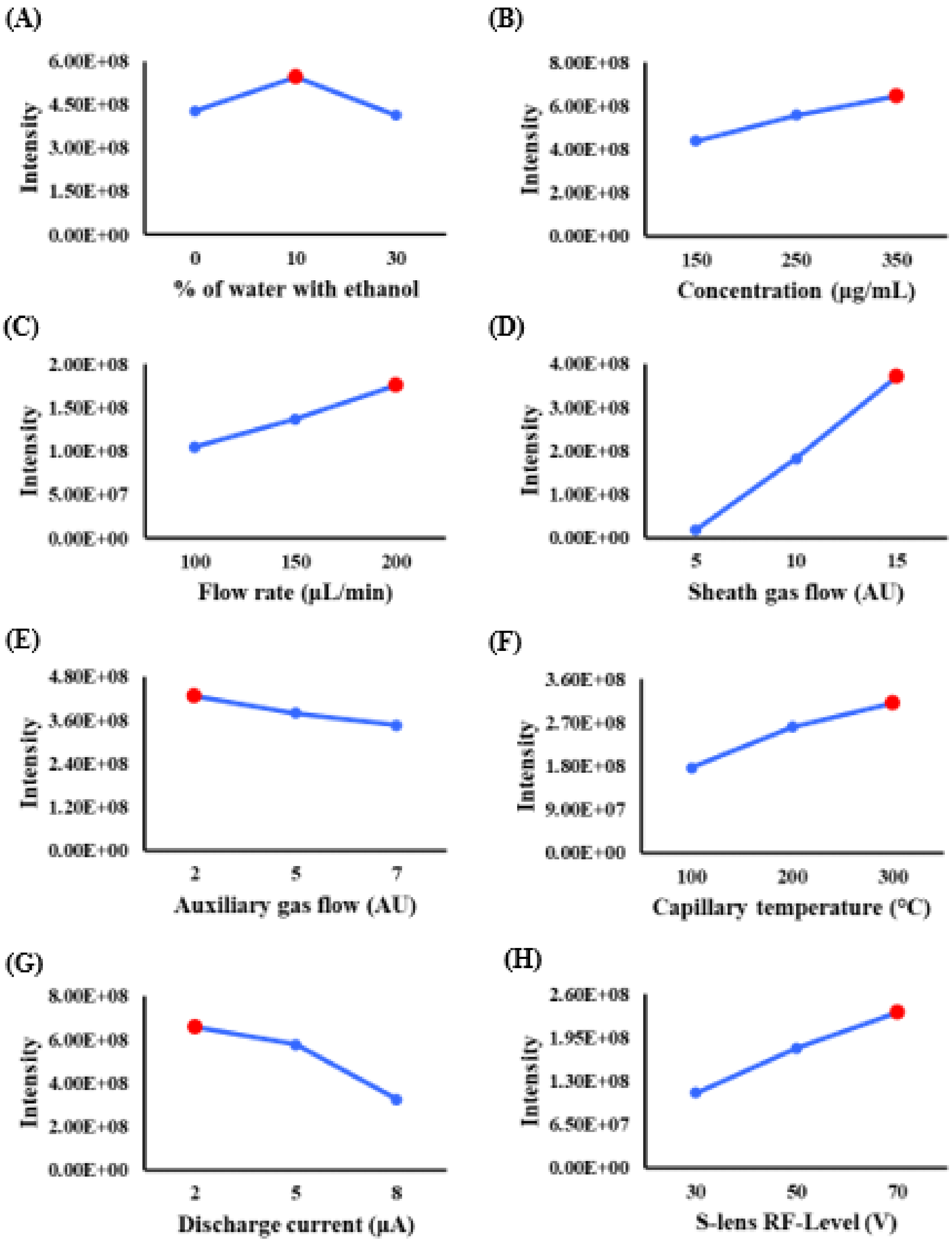

Figure S11: Optimization of (–) mode atmospheric pressure chemical ionization mass spectrometry (APCI-MS) operating parameters.

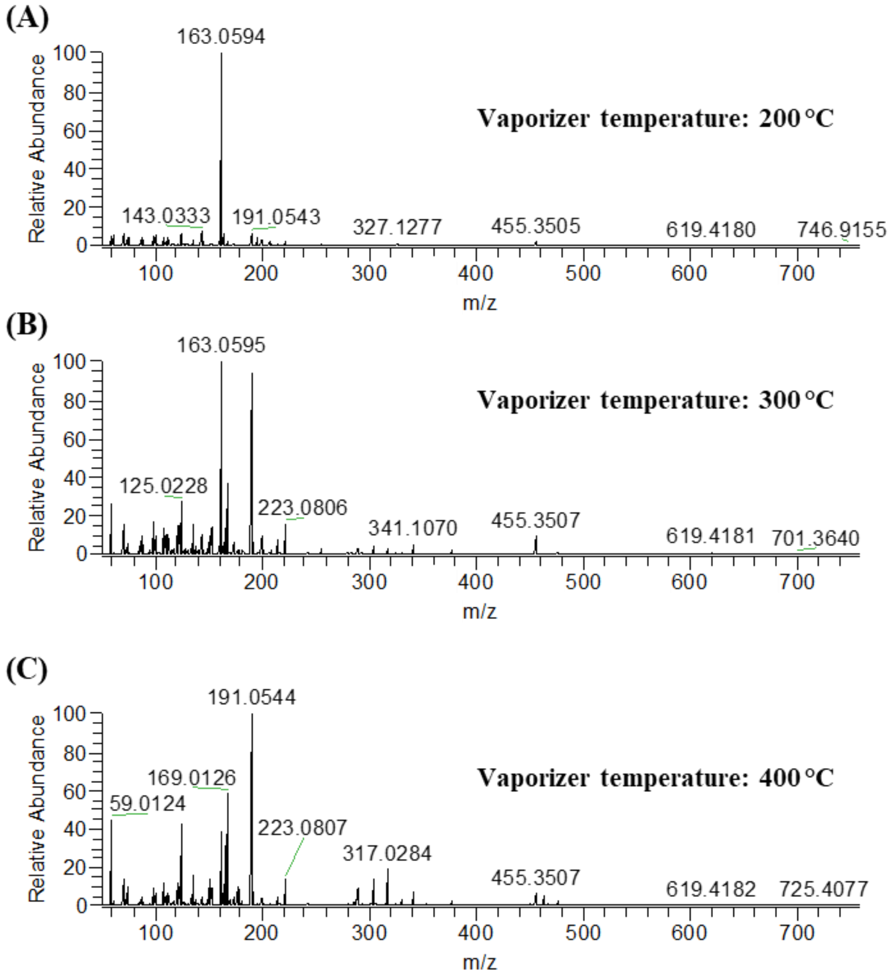

Figure S12: (–) mode atmospheric pressure chemical ionization (APCI) mass spectra at different vaporizer temperatures.

Table S2. Optimized operational conditions for negative-mode electrospray ionization mass spectrometry (ESI–MS).

parameters	values considered for optimization	optimized value
composition of % of water with ethanol	0, 10, 30, 50	10
concentration, µg/mL	50, 100, 150, 200, 250, 300, 350, 400	350
flow rate, µL/min	10, 20, 30	20
sheath gas flow, AU	4, 6, 8	6
auxiliary gas flow, AU	2, 4, 6	2
capillary temperature, °C	200, 300, 400	400
spray voltage, kV	3.2, 3.6, 4, 4.4	4
S-lens RF level, V	10, 30, 50, 60	50

Table S3. Optimized operational conditions for (–) mode atmospheric pressure chemical ionization mass spectrometry (APCI-MS).

Parameters	Values considered for optimization	Optimized value
Composition of % of water with ethanol	0, 10, 30	10
Concentration, µg/mL	150, 250, 350	350
Flow rate, µL/min	100, 150, 200	200
Sheath gas flow, AU	5, 10, 15	15
Auxiliary gas flow, AU	2, 5, 7	2
Capillary temperature, °C	100, 200, 300	300
Vaporizer temperature, °C	200, 300, 400	400
Discharge current, µA	2, 5, 8	2
S-lens RF-level, V	30, 50, 70	70
