# Supplementary material for: Metabolite Profiling of Manilkara zapota L. Leaves by High-Resolution Mass Spectrometry Coupled with ESI and APCI and In Vitro Antioxidant Activity, α-Glucosidase, and Elastase Inhibition Assays
Source: Int J Mol Sci. 2020 Dec 24;22(1):132. doi: 10.3390/ijms22010132 (PMC7795549; doi:10.3390/ijms22010132)
Supplement: Supplementary file 1 [file ijms-22-00132-s001.zip › Supplementary-materials_ZLE_Final.docx]

Metabolite Profiling of *Manilkara zapota* L. Leaves by High-Resolution Mass Spectrometry Coupled with ESI and APCI and in Vitro Antioxidant Activity, α-Glucosidase, and Elastase Inhibition Assays

Syful Islam ^1,2,†^, Md Badrul Alam ^3,4,†^, Hyeon-Jin Ann ^3^, Ji-Hyun Park ^3^, Sang-Han Lee ^3,4,5,^* and Sunghwan Kim ^1,6,^*

^1^ Department of Chemistry, Kyungpook National University, Daegu 41566, Korea; msi412@yahoo.com

^2^ Department of Environment, Munshiganj District Office, Munshiganj-1500, Bangladesh

^3^ Department of Food Science and Biotechnology, Kyungpook National University, Daegu 41566, Korea; mbalam@knu.ac.kr (M.B.A.); jiny345@knu.ac.kr (H.-J.A.); wlgus6744@knu.ac.kr (J.-H.P.)

^4^ Food and Bio-Industry Research Institute, Inner Beauty/Antiaging Center, Kyungpook National University, Daegu 41566, Korea

^5^ knu BnC, Daegu 41566, Korea

^6^ Mass Spectrometry Converging Research Center and Green-Nano Materials Research Center, Kyungpook National University, Daegu 41566, Korea

***** Correspondence: sang@knu.ac.kr (S.-H.L.); sunghwank@knu.ac.kr (S.K.)

† These authors have equal contribution

**Supplementary materials:**

**
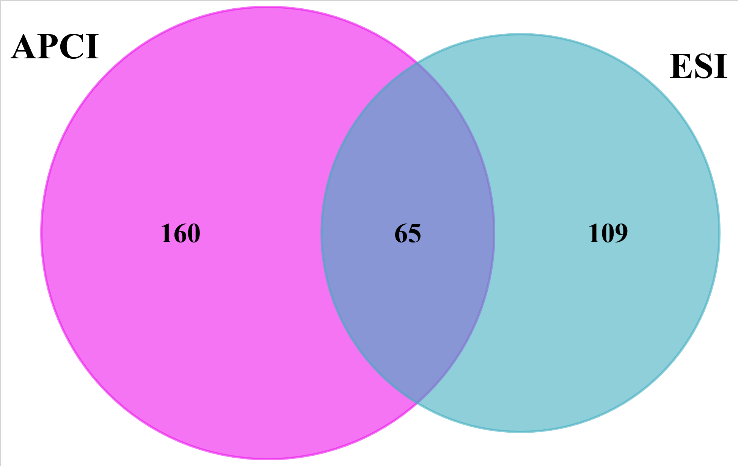
**

**Figure S1:** Venn diagram showing the overlapping peaks detected in (–) mode electrospray ionization (ESI) and atmospheric pressure chemical ionization (APCI) mass spectrometry (MS).


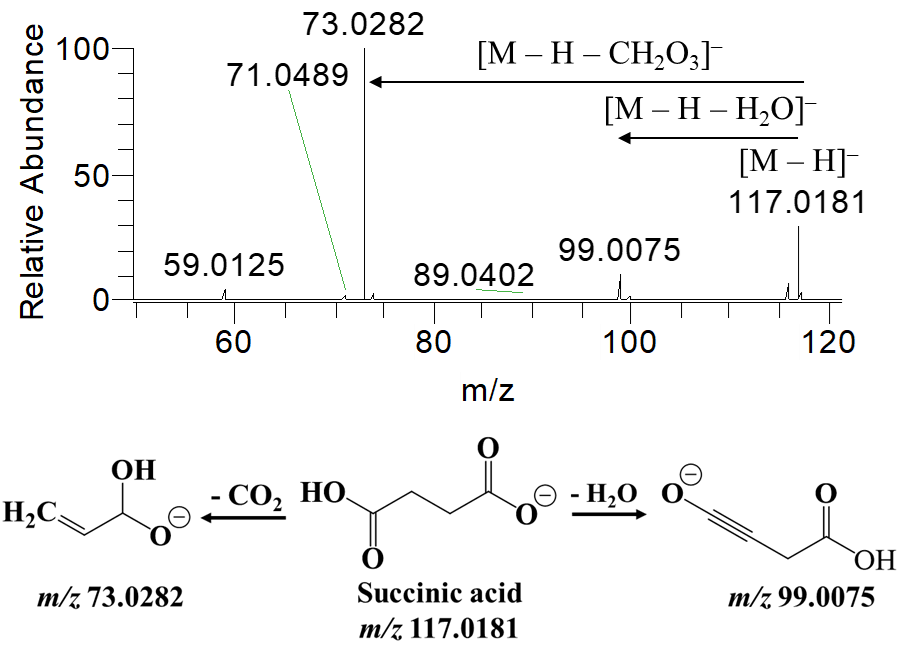


**Figure S2:** (–) mode electrospray ionization tandem mass spectrometry (ESI MS/MS) fragmentation pattern of succinic acid.


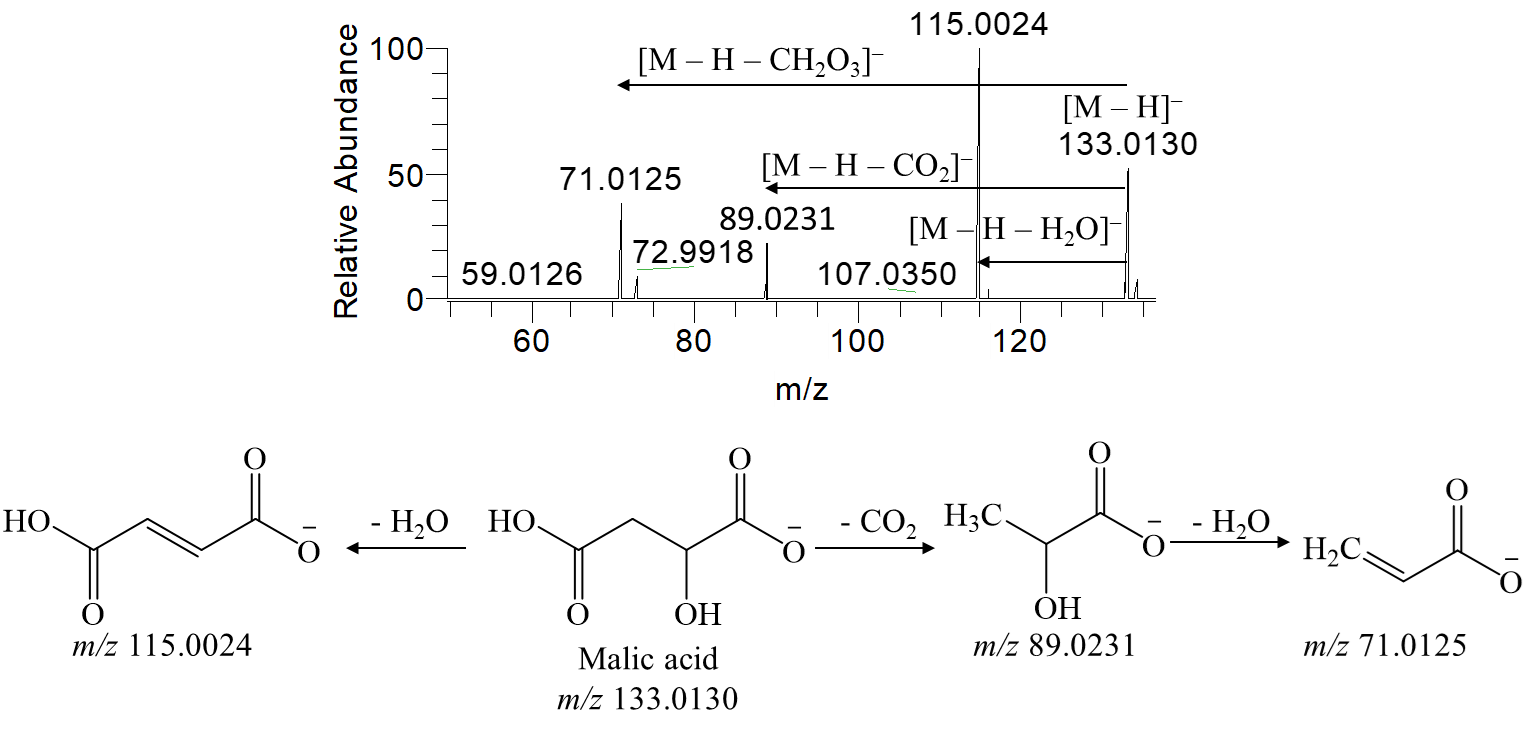


**Figure S3:** (–) mode electrospray ionization tandem mass spectrometry (ESI MS/MS) fragmentation pattern of malic acid.


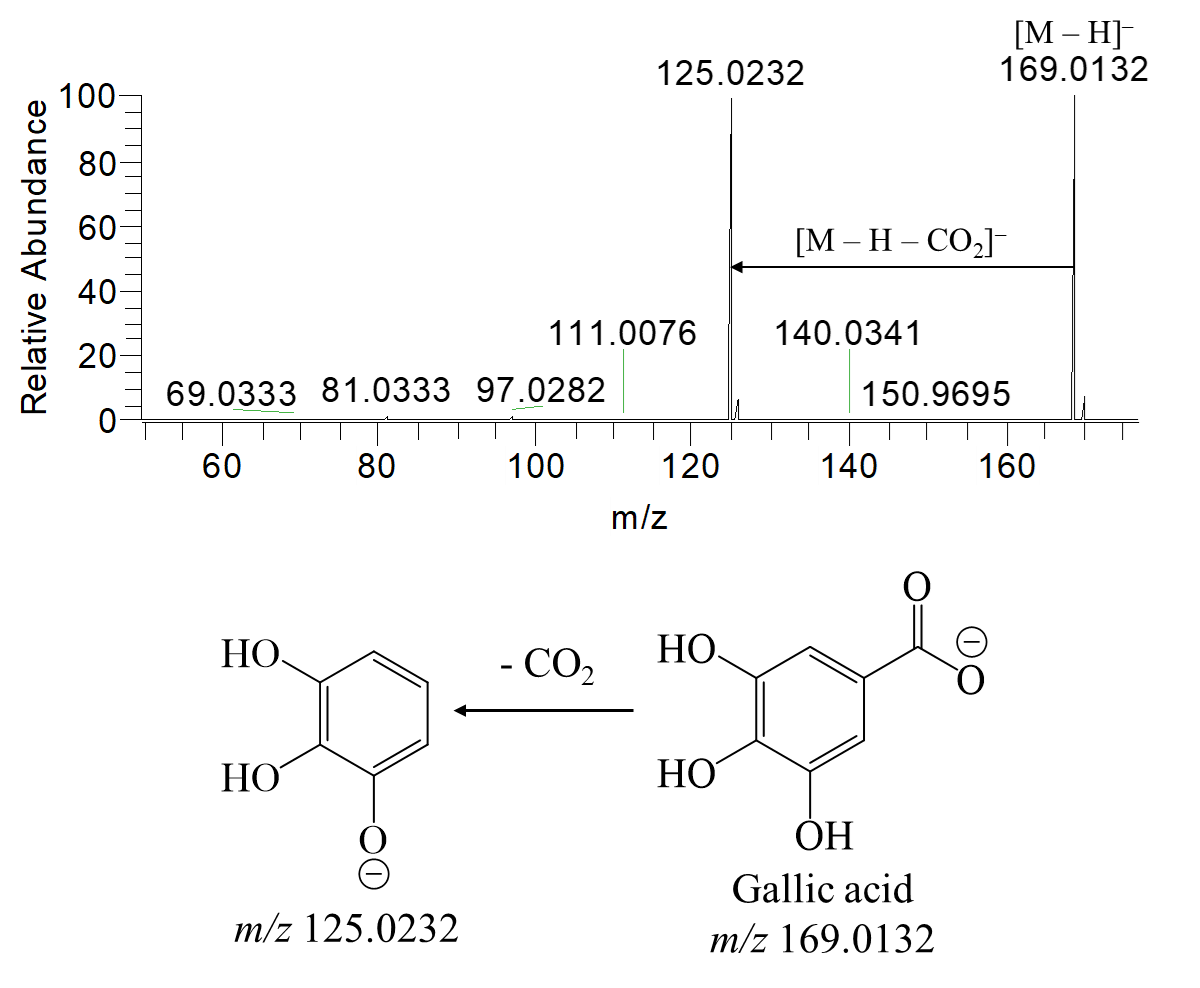


**Figure S4:** (–) mode electrospray ionization tandem mass spectrometry (ESI MS/MS) fragmentation pattern of gallic acid.


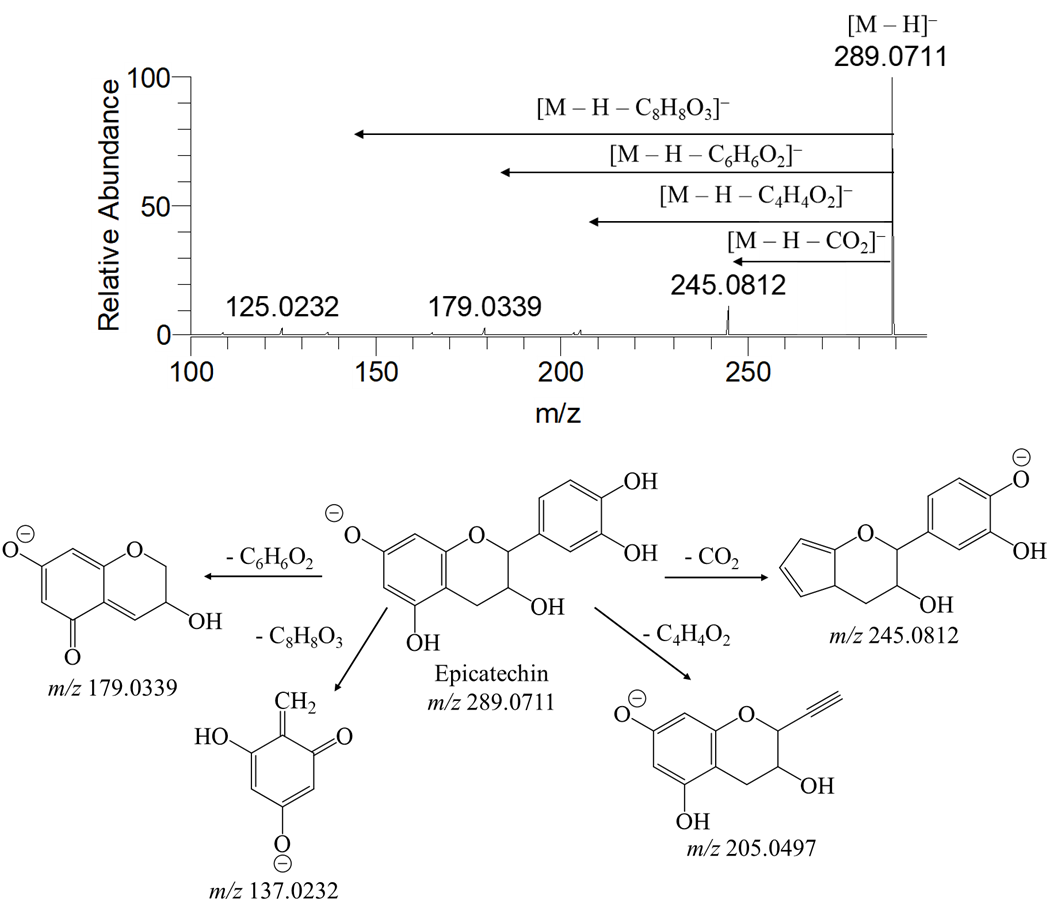


**Figure S5:** (–) mode electrospray ionization tandem mass spectrometry (ESI MS/MS) fragmentations of epicatechin.


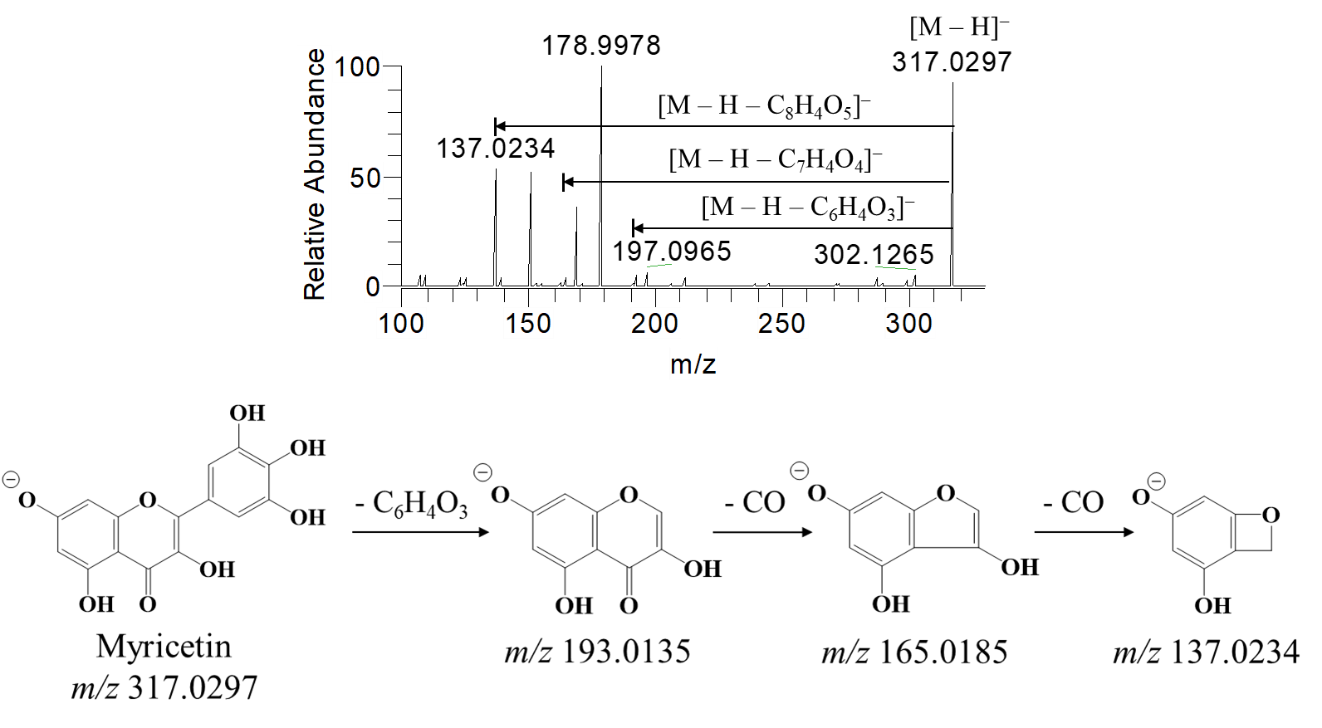


**Figure S6:** (–) mode atmospheric pressure chemical ionization tandem mass spectrometry (APCI MS/MS) fragments of myricetin.


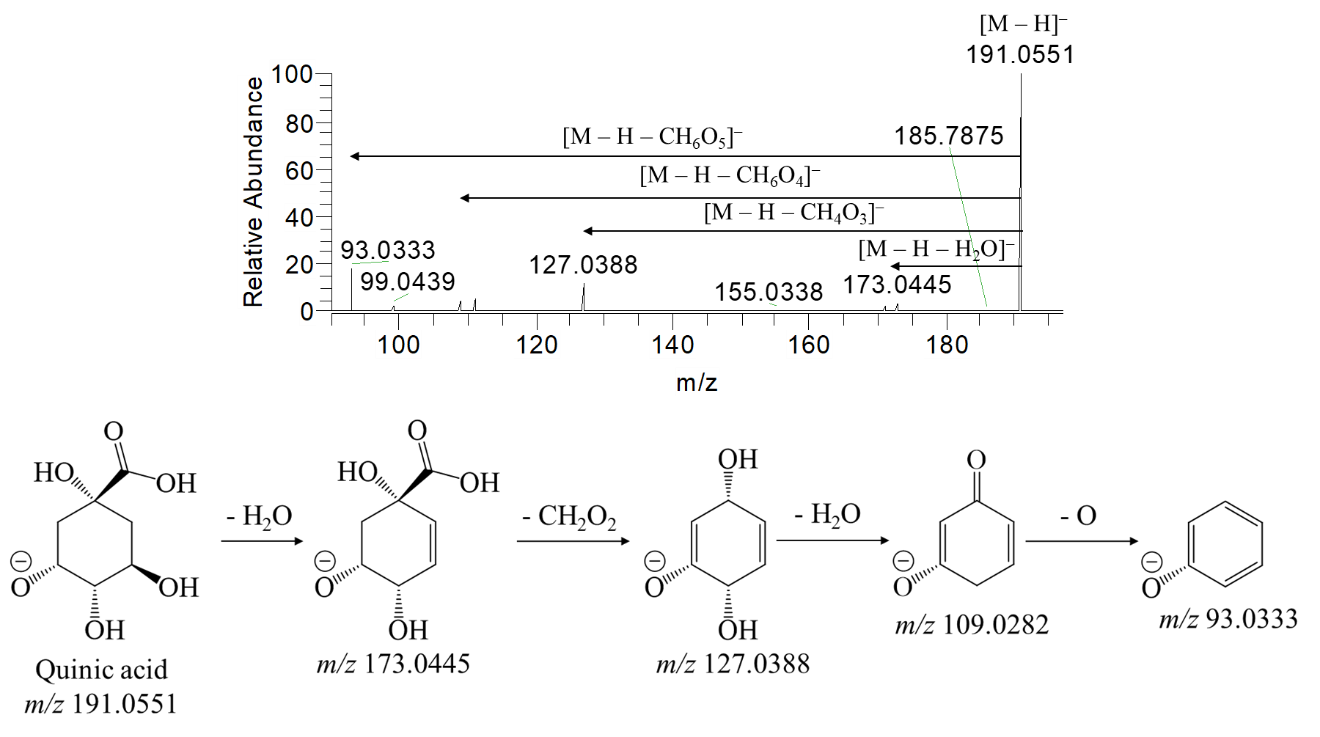


**Figure S7:** (–) mode electrospray ionization tandem mass spectrometry (ESI MS/MS) fragments of quinic acid.


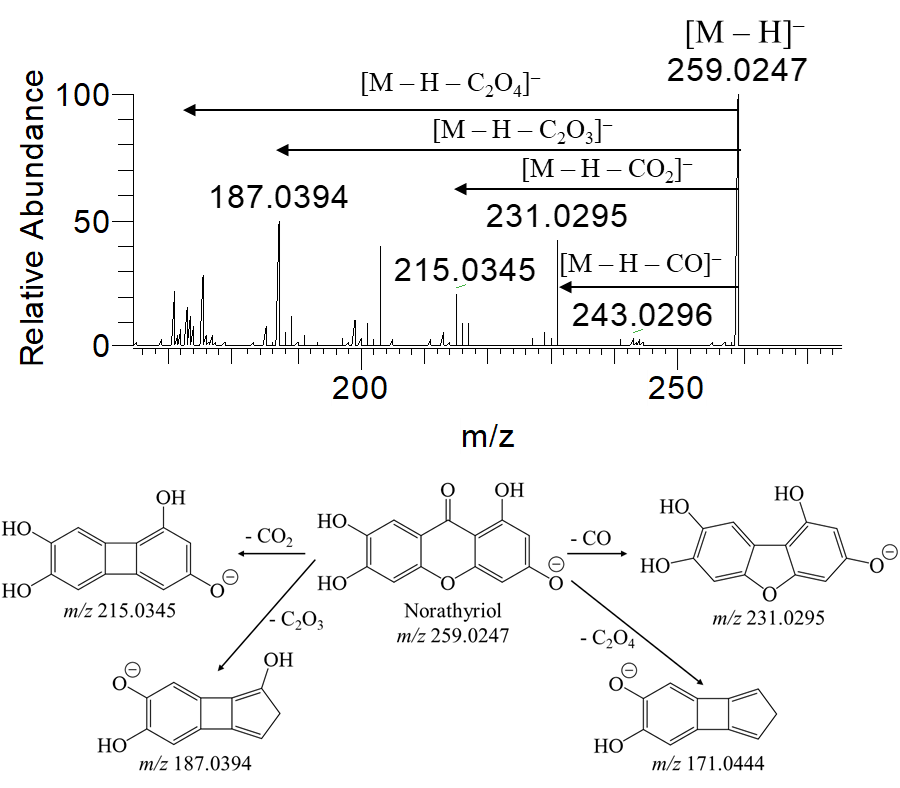


**Figure S8:** (–) mode atmospheric pressure chemical ionization tandem mass spectrometry (APCI MS/MS) fragments of norathyriol.


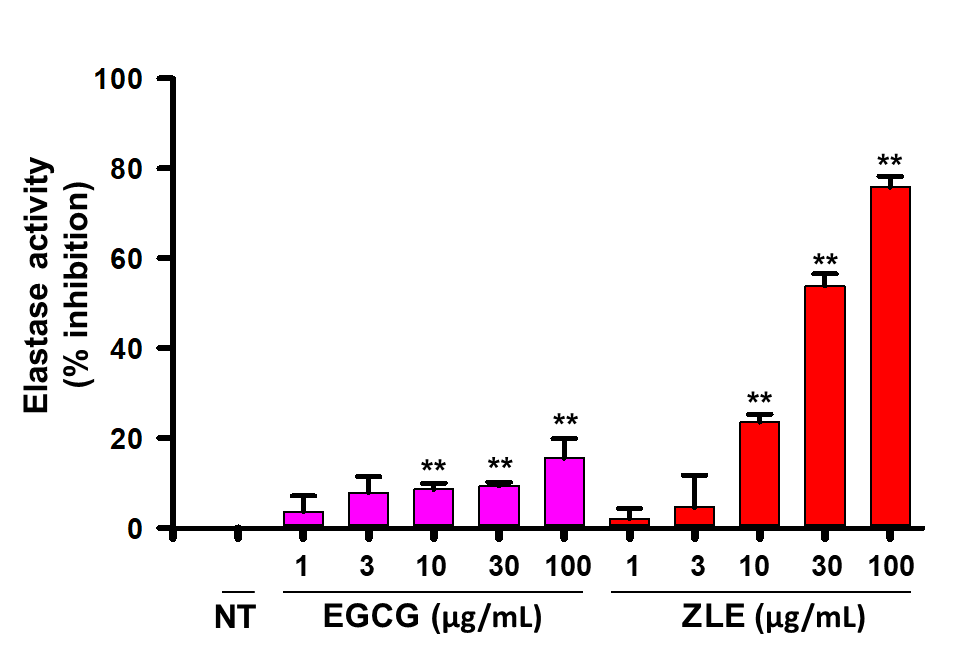


**Figure S9:** Elastase-inhibition activities of *Manilkara zapota* leaves ethanol extracts (ZLE).


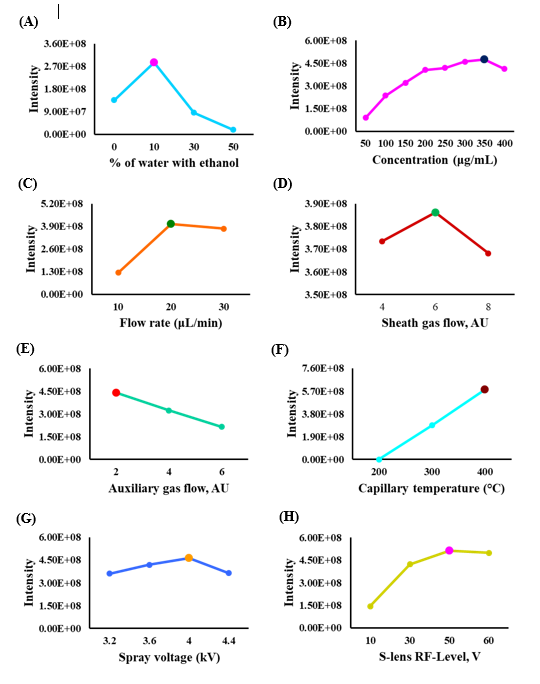


**Figure S10:** Optimization of negative-mode electrospray ionization mass spectrometry (ESI–MS) operating parameters.


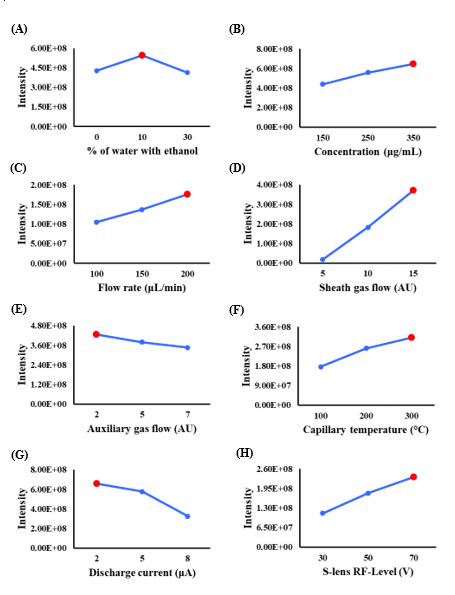


**Figure S11:** Optimization of (–) mode atmospheric pressure chemical ionization mass spectrometry (APCI-MS) operating parameters.


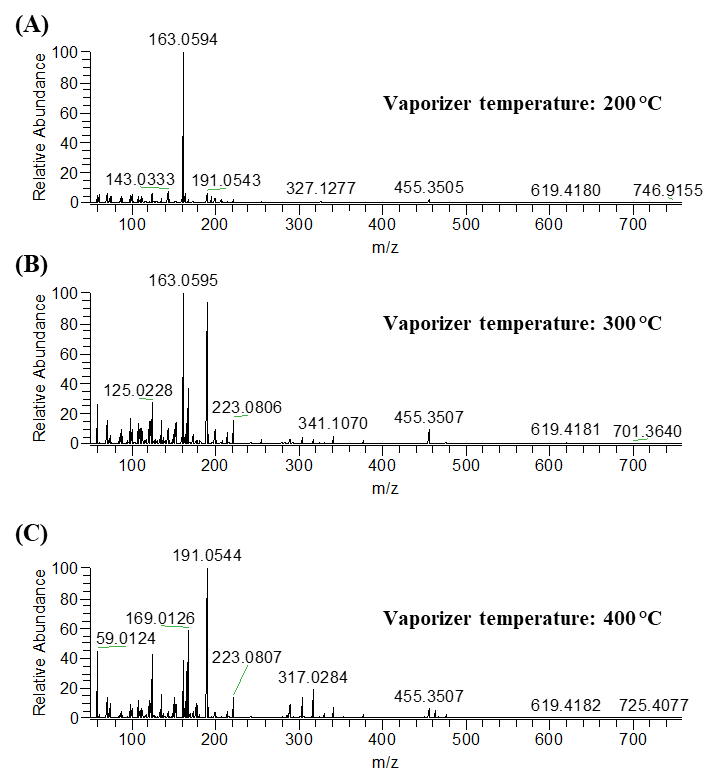


**Figure S12:** (–) mode atmospheric pressure chemical ionization (APCI) mass spectra at different vaporizer temperatures.

**Table S2.** Optimized operational conditions for negative-mode electrospray ionization mass spectrometry (ESI–MS).

| **parameters** | **values considered for optimization** | **optimized value** |
| --- | --- | --- |
| composition of % of water with ethanol | 0, 10, 30, 50 | 10 |
| concentration, μg/mL | 50, 100, 150, 200, 250, 300, 350, 400 | 350 |
| flow rate, μL/min | 10, 20, 30 | 20 |
| sheath gas flow, AU | 4, 6, 8 | 6 |
| auxiliary gas flow, AU | 2, 4, 6 | 2 |
| capillary temperature, °C | 200, 300, 400 | 400 |
| spray voltage, kV | 3.2, 3.6, 4, 4.4 | 4 |
| S-lens RF level, V | 10, 30, 50, 60 | 50 |

**Table S3.** Optimized operational conditions for (–) mode atmospheric pressure chemical ionization mass spectrometry (APCI-MS).

| **Parameters** | **Values considered for optimization** | **Optimized value** |
| --- | --- | --- |
| Composition of % of water with ethanol | 0, 10, 30 | 10 |
| Concentration, μg/mL | 150, 250, 350 | 350 |
| Flow rate, μL/min | 100, 150, 200 | 200 |
| Sheath gas flow, AU | 5, 10, 15 | 15 |
| Auxiliary gas flow, AU | 2, 5, 7 | 2 |
| Capillary temperature, °C | 100, 200, 300 | 300 |
| Vaporizer temperature, °C | 200, 300, 400 | 400 |
| Discharge current, μA | 2, 5, 8 | 2 |
| S-lens RF-level, V | 30, 50, 70 | 70 |
